# Complete Genome Sequence of a Rhabdovirus Strain from *Culex* Mosquitos Collected in Southern Switzerland

**DOI:** 10.1128/MRA.01234-20

**Published:** 2021-01-07

**Authors:** Jakub Kubacki, Isabelle Hardmeier, Weihong Qi, Eleonora Flacio, Mauro Tonolla, Cornel Fraefel

**Affiliations:** a Institute of Virology, University of Zurich, Zurich, Switzerland; b Functional Genomics Center Zurich, Zurich, Switzerland; c Laboratory of Applied Microbiology, University of Applied Sciences and Arts of Southern Switzerland, Manno, Switzerland; KU Leuven

## Abstract

We report here the full-length genome sequence of a rhabdovirus strain detected in a pool of 21 Culex pipiens and Culex torrentium mosquitos collected in southern Switzerland. The genome has a length of 11,914 nucleotides and encodes five major putative open reading frames.

## ANNOUNCEMENT

*Rhabdoviridae* is an ecologically highly diverse virus family with members detected in a broad range of hosts, e.g., mammals, fish, plants, and insects (1, 2). Arthropods such as mosquitos, midges, and ticks can act as vectors, reservoirs, and hosts for some rhabdoviruses (3). The rhabdovirus genome is a single-stranded RNA genome of negative polarity that ranges from 11 to 15 kb, depending on the virus species, and encodes five major open reading frames (ORFs).

**FIG 1:**
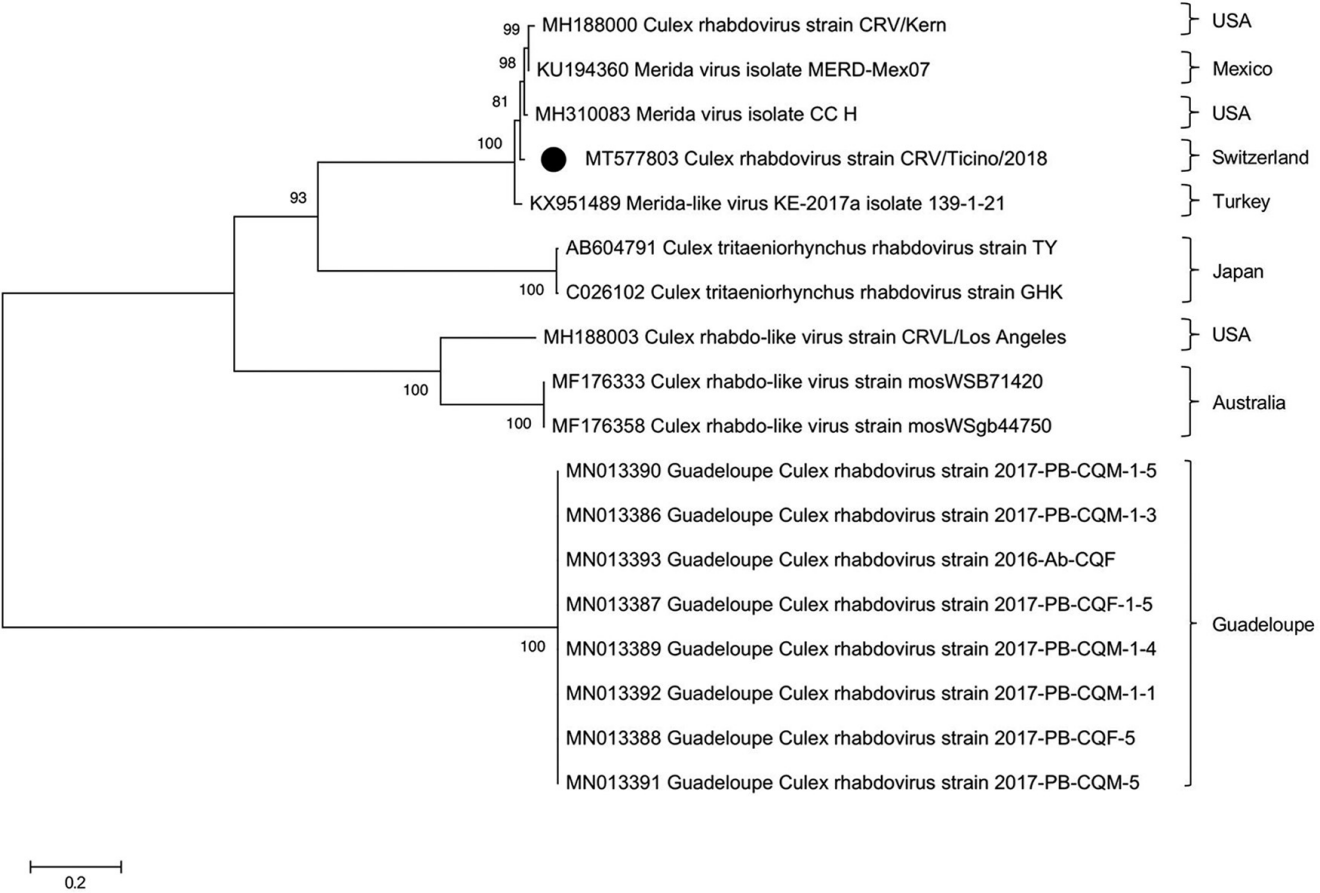
Phylogenetic reconstruction based on the RNA-dependent RNA polymerase (RdRp) protein and the location where *Culex* mosquitoes were captured. The phylogenetic tree was constructed by the maximum likelihood method using MEGA X software with 100 bootstrap replicates. The RdRp sequence of the rhabdovirus genome identified in this study is marked with a black dot.

Here, we present the full genome sequence of a rhabdovirus strain from *Culex* mosquitos collected in southern Switzerland in 2018, particularly in the municipality of Gambarogno. In total, 21 *C. pipiens* and *C. torrentium* mosquitos were pooled and homogenized in 500 µl of phosphate-buffered saline (PBS) using the TissueLyser II (Qiagen, Germany) at 20 Hz for 2 min. Total RNA was extracted using the QIAamp viral RNA minikit, according to the manufacturer’s instructions (Qiagen). After the RNA was reverse transcribed using random primers with a known 20-nucleotide (nt) tag sequence and the RevertAid First Strand H minus cDNA synthesis kit (Thermo Fisher, Switzerland), sequence-independent single-primer amplification was performed (4). The libraries were prepared for next-generation sequencing (NGS) using the NEBNext Ultra II DNA library preparation kit and NEBNext multiplex oligos for the Illumina barcoding kit, according to the manufacturer’s manual (New England BioLabs, Switzerland). A paired-end NGS run of 2 × 150-nt read length was performed on an Illumina NextSeq 500 sequencing system using a high-output flow cell at the Functional Genomics Center Zurich (Switzerland). In total, 2.58 million raw reads were sequenced, and then quality control was performed using FastQC (version 0.11.7), reads were trimmed from PCR primers, sequencing adaptors and low-quality ends were trimmed by Trimmomatic (version 0.36), and contigs were assembled using MEGAHIT (version 1.1.3) with settings as described previously (5). Assembled contigs were compared against the NCBI nt database (ftp://ftp.ncbi.nlm.nih.gov/blast/db/) using blastn (version 2.6.0+) and visualized and manually confirmed using the SeqMan Pro software version 17 (DNAStar [Lasergene, USA]). From 62,730 sequencing reads, a single consensus sequence of 11,914 nt with high similarity to genomes of viruses from *Rhabdoviridae* was generated. Specifically, alignment of the consensus sequence revealed genetic similarity to the Merida virus isolate MERDV-Mex07 and the *Culex* rhabdovirus strain CRV/Kern, with 91.62% and 91.29% identity, respectively (Fig. 1) (6, 7). The genome of this rhabdovirus strain (CRV/Ticino) has a GC content of 49% and contains five major ORFs as specified in the GenBank entry. The amino acid sequences of nucleoprotein (N), matrix protein (M), glycoprotein (G), and RNA-dependent RNA polymerase (L) have 95.6%, 95.7%, 98.6%, and 97.3% identity, respectively, to MERDV. The phosphoprotein (P) has 86.7% identity to CRV/Kern. This is the first report of a rhabdovirus genome detected in *Culex* mosquitos collected in Switzerland, thereby expanding the knowledge on rhabdovirus diversity circulating in the environment. However, the original host of the virus cannot be determined by sequencing of mosquito samples, as it may have been acquired from feeding.

### Data availability.

This sequence has been deposited in GenBank under the accession number MT577803. The raw metagenomic data have been deposited in the NCBI Sequence Read Archive under the accession number SRR12153462.
